# Analysis of oral microbiota in non-vital teeth and clinically intact external surface from patients with severe periodontitis using Nanopore sequencing: a case study

**DOI:** 10.1080/20002297.2023.2185341

**Published:** 2023-03-03

**Authors:** Alessio Buonavoglia, Francesco Pellegrini, Gianvito Lanave, Georgia Diakoudi, Maria Stella Lucente, Fausto Zamparini, Michele Camero, Maria Giovanna Gandolfi, Vito Martella, Carlo Prati

**Affiliations:** a Department of Biomedical and Neuromotor Sciences, Dental School, University of Bologna, Bologna, Italy; b Department of Veterinary Medicine, University of Bari, Valenzano, Bari, Italy

**Keywords:** Microbiota, root canal, intact teeth, periodontitis, Nanopore

## Abstract

Periodontal diseases include a wide range of pathological conditions, damaging the supporting structures of the teeth. Origin and propagation of periodontal disease is believed to be caused by dysbiosis of the commensal oral microbiota. The aim of this study was to evaluate the presence of bacteria in the pulp cavity of teeth with severe periodontal disease with clinically intact external surface. Periodontal (P) and endodontic (E) tissue samples of root canals from six intact teeth of three patients were sampled for analysis of microbial population using Nanopore technology. *Streptococcus* was the predominant genus in E samples. *Porphyromonas* (33.4%, *p* = 0.047), *Tannerella* (41.7%, *p* = 0.042), and *Treponema* (50.0%, *p* = 0.0064) were significantly more present in P than in E samples. Some samples (E6 and E1) exhibited a remarkable difference in terms of microbial composition, whilst *Streptococcus* was a common signature in samples E2 to E5, all which were obtained from the same patient. In conclusion, bacteria were identified on both the root surface and the root canal system, thus demonstrating the possibility of bacteria to spread directly from the periodontal pocket to the root canal system even in the absence of crown’s loss of integrity.

## Introduction

Although periodontal and endodontic tissues are prone to different pathologies, there are strict anatomical correlations through lateral and accessory canals and the apical foramen and dentinal tubules [1], favoring bacterial migration among contiguous tissues [1–3]. Bacterial biofilms associated with endodontic or periodontal infections are similar, confirming these pathways of migration [4] Endo-periodontal lesions (EPL) are bacterial infectious diseases that affect both periodontal and endodontic tissues of the same tooth, with periodontal tissue damage and pulp inflammation/necrosis, defined by a pathological communication between the pulpal and periodontal tissues [5].

The common pathologic communication between these tissues can occur by a carious or traumatic lesion that affects the pulp and, secondarily periodontium between apical foramen, accessory canals, and dentinal tubules [6].

According to the classification of American Academy of Periodontology criteria, EPL occur in either an acute or a chronic form and are classified according to signs and symptoms that have direct impact on their prognosis and treatment. The primary signs are deep periodontal pockets extending to the root apex and/or negative/altered response to pulp vitality tests. Other signs/symptoms may include radiographic evidence of bone loss in the apical or furcation region, pain, suppuration, tooth mobility, sinus tract, and crown and/or gingival colour alterations. EPL can be associated with a root damage, generally determined by traumatic and/or iatrogenic factors that may include root perforation, fracture/cracking, or external root resorption. These conditions drastically impair the prognosis of the involved tooth. EPL without root damage are distinguished in two categories based on the presence of periodontitis. EPL are graded into three levels depending on morphology and width of the periodontal pocket. Grade 1 presents narrow deep periodontal pocket in 1 tooth surface, Grade 2 a wide deep periodontal pocket in the tooth surface and Grade 3 a deep periodontal pockets in >1 tooth surface. EPL Grade 3 with root damage have the worst prognosis [5].

When it comes to pulpal pathology of periodontal origins, the matter is more controversial, especially in teeth that present only a periodontal pathology without root damages and/or coronal leakages [7,8].

Massive periodontal destruction can secondarily affect the root canal system with dissemination of the inflammation, which can result in pulp necrosis [9]. It is still unclear if bacteria can reach the root canal system, or if inflammation is only due to their metabolic products. Overall, how bacteria can reach the root canal system from periodontal tissue is largely unclear. The aim of this study was to evaluate if in non-vital teeth with severe periodontal disease not reaching the apex root segment and with clinically intact external surface there is presence of bacteria in the endodontic space.

## Materials and methods

### Case study

Study participants were three patients (two female and one male patient; mean age, 51 years) with chief complaint of severe lower anterior teeth mobility and severe periodontal compromission hindering any periodontal or conservative treatment. As control case, a patient (#1) referred with chief complaint due to lower third molar pericoronitis and indication for surgical extraction was recruited.

The exclusion criteria for this study were antibiotic therapy administered up to 3 months before tooth extraction, systemic diseases, and pregnancy. Another exclusion criterium was evaluating that periodontal pockets could not reach the apical root segment. Moreover, inclusion criteria were teeth affected by EPL without clinically and radiographically identified caries lesions, cracks/fractures and/or restorations.

The patients involved in the research signed a formal written informed consent form.

EPL diagnosis was performed with periodontal probing that evaluate an average clinical attachment loss ≥5 mm on all root’s surfaces and radiographic analyses on periapical bidimensional radiographies using paralleling technique confirming bone loss extending to mid-third of root and beyond; moreover, periapical radiolucency was not observed. None of these teeth presented periodontal pockets reaching the apex. Teeth presented Grade 2 mobility with percussion and palpation sensitivity. In addition, thermal and electric pulp sensibility tests were performed returning non-responsivity and thus confirming pulpar necrosis. Thermal pulp test was performed with a #2 cotton pellet sprayed and fully saturated with 1,1,1,2-tetrafluoroethane and placed at the middle third of the buccal tooth’s surface of the clinical crown for at least 20 s. Cold pulp testing was selected as pulp sensibility test which is able to evaluate vital (specificity = 0.84) and non-vital (sensitivity = 0.87) teeth [10–12]. No other EPL signs/symptoms (sinus tract, spontaneous pain, periapical radiolucency, suppuration) were observed.

Moreover, on intraoral inspection using loupes for magnification 4.0× and radiographic evaluation, the teeth did not present clinically identified caries lesions, cracks, fractures nor previous conservative restorations. The final diagnosis for all teeth was of EPL without root damage of Grade 3, according to the classification from the American Academy of Periodontology criteria. The sampled teeth clinically appeared without defects, decay, or restorations and were affected by severe periodontitis (Stage IV) according to the classification of American Academy of Periodontology criteria [5]. Control sample consisted of an intact lower third molar without carious and periodontal pathologies, surgically extracted because affected by pericoronitis. All teeth affected by EPL were single-rooted (*N* = 5 lower incisors and *N* = 1 lower canine).

A total of 12 clinical samples of the study group were collected from periodontal (P) (*n* = 6) and endodontic (E) (*n* = 6) tissue samples of root canals from six intact teeth of three patients (P1 and E1 from patient #1, P2 to P5 and E2 to E5 from patient #2, and P6 and E6 from patient #3).

All teeth were single-rooted (lower incisors and lower canine).

The study was performed in agreement with the ethical guidelines of the Declaration of Helsinki laid down in the 1964 and its later amendments or comparable ethical standards. The Ethics Committee of Azienda Unità Sanitaria Locale of Bologna approved this study with authorization no. 844-2021-OSS-AUSLBO-21160-ID 3118-Parere CE-AVEC-ENDO-MICROBIOTA 09/2021.

### Root canal sampling

Non-surgical periodontal treatment was applied using ultrasonic tips to remove supragingival dental biofilms and pre-operative mouthwash with chlorhexidine 0.20% for 60 s to reduce bacterial load.

Subsequently, the teeth were anesthetized using articaine with adrenaline 1:100.000 (Septodont, Saint-Maur-des-Fossés, France). Sindesmotomy and luxation were performed with a rounded periosteal elevator; extraction was gently performed with dental forceps and tooth was positioned in a sterile tube (Eppendorf AG, Hamburg, Germany). An accurate alveolar toilette was performed with mechanical debridement of granulation tissue and subsequent intra-alveolar irrigation with sterile saline solution rinse. A resorbable collagen sponge (Septodont, Saint-Maur-des-Fossés, France) was positioned in dental socket and a criss-cross non-resorbable suture was performed to favor haemostasis. Only for the extraction of the third molar, a mucoperiosteal flap was executed without ostectomy.

After extraction, all teeth were visually examined using loupes for magnification 4.0× to exclude caries lesions, cracks, fractures nor previous conservative restorations.

Sampling procedures were carried out immediately after extraction using sterile gloves and sterile materials/instruments. In detail, P samples were collected using a sterile swab to scrub on root surface, chiefly where subgingival calculus was visible; subsequently, swabs were inserted in sterile tubes (Aptaca Spa, Canelli AT, Italy) provided with Stuart transport medium and stored at −80°C until use.

Subsequently the crown was disinfected with 2.5% sodium hypochlorite solution (NaOCl) (Niclor 2.5, Ogna, Maggiò, Italy) for 30 s [8,13]. The NaOCl solution was inactivated with 5% sodium thiosulfate to avoid interference for diffusion of NaOCl in root canal system during cavity access preparation and bacteriological sampling.

To control the sterility of the operating field, two sterile cotton pellets were brushed on the disinfected tooth crown and transferred to a tube containing transport fluid. If bacterial growth was detected within 72 h at 37°C in laboratory incubator, the sample of the root canal was excluded from the study.

Preparation of the access cavity was performed using a sterile high-speed diamond bur (Maillefer, Ballaigues, Switzerland) under sterile saline solution flow. Before the pulp chamber was exposed, cleaning of the tooth was repeated as previously described. All the remaining pulpal tissues observed were evaluated clinically as non-bleeding, fibrotic and without chromatic aspects (red or pink colouring) traceable to vital pulp. Moreover, pulp space appeared more or less empty, to confirm clinical diagnosis of pulpal necrosis.

After gentle irrigation with sterile saline solution, a sterile #10 K-type stainless hand file (Maillefer, Ballaigues, Switzerland) was introduced into the canal at the tooth apex level. In the control case (lower molar), E samples were collected from the largest root canal (distal root). Working length was previously calibrated on clinical tooth’s length to stop K-file and paper points at the level of the tooth apex level. These procedures were carried out by means of a visual inspection using magnification loupes to prevent the crossing of the apex by K-file and paper points.

Following gently scraping with instrumentation alongside the root canal walls with a sterile #10 K-type stainless hand file (Maillefer, Ballaigues, Switzerland) to disperse bacteria in the medium, sterile paper points #15 (Dentsply-Maillefer, Ballaigues, Switzerland) were positioned in the canals for 60 s, to collect ‘E’ samples in sterile tubes (Eppendorf AG, Hamburg, Germany), subsequently stored at − 80°C until use. Every procedure was executed using new sterile gloves.

### DNA extraction

Paper point samples were immersed in a 2-mL Eppendorf safe-lock tube containing Dulbecco’s Minimal Essential Medium (DMEM). Subsequently, samples were homogenized by Tissue Lyser (Qiagen GmbH, Hilden, Germany) at 30 Hz for 5 min. Homogenized samples were centrifuged at 10,000 × *g* for 3 min. A total of 200 µL of supernatants were subjected to DNA extraction using DNeasy PowerSoil PRO kit (Qiagen S.p.A., Milan, Italy) according to manufacturer’s instructions. Negative controls of extraction (DMEM and reagents from extraction kits) were used at the same time as samples, to check for the presence of possible contamination during the extraction steps. To assess for bias in extraction and/or sequencing, commercially available mock community control composed of three Gram-negative and five Gram-positive bacteria with a range of GC content was used. Mock community DNA obtained by pooling DNA extracted from pure cultures (ZymoBIOMICS Catalog #D6306) was used as the input DNA.

### *PCR amplification of* 16SrDNA *gene and Nanopore* sequencing

A PCR protocol was performed on DNA extracts to amplify the full-length (1500bp) sequence of the *16S rRNA* gene using universal primers [14] and TaKaRa LA TaqTM kit (Takara Bio Europe S.A.S., Saint-Germain-en-Laye, France). Afterwards, the 16S barcoding kit SQK-RAB204 (Oxford Nanopore Technologies, ONT, Oxford UK) was used to prepare libraries which were purified by Agencourt AMPure XP magnetic beads (Beckman Coulter™), pooled and sequenced using MinION flongle Flow cell FLO-FLG001, version R9.4.1 adapted on the MinION- Mk1C device (ONT, UK) for 24 h.

### Data analysis

FastQ MinION files were uploaded on the online EPI2ME platform (https://epi2me.nanoporetech.com/) and analyzed by the Fastq 16S 2021.09.09 (Metrichor Agent, ONT) workflow setting the following parameters: quality score 10, minimum length filter of 1500 bases, and BLAST E-value of 0.01.

Taxonomy was obtained through interrogation of the NCBI database non-redundant using BLAST with a minimum horizontal coverage of 30% and a minimum accuracy of 77% as default parameters. Reads data obtained were organized in Microsoft Office Excel. Only Taxa scoring a ≥ 0.1% relative abundance in samples were considered and thus analyzed using the ‘Plotly.py’ open-source library for Python 3.7.9 [15]. The computed data were then represented as interrogable BarPlot charts.

In addition, ‘Krona’ visualization tool was employed to organize and display the communities at a species level in multi-layered pie [16]. Multiple comparisons of the bacterial sequence reads obtained in the P and E tissue samples was compared using Kruskal–Wallis test with Dunn test as post hoc test. Moreover, categorical dichotomous data (P and E tissue samples and presence/absence of bacteria in samples) were described as counts and percentages and evaluated by Fisher’s exact test. Statistical analyses were performed using the freely available online tool EZR [17] for personal computers. A *p*-value <0.05 was considered for statistical significance.

### Diversity indexes

Statistical analyses were performed with R v.4.1.3 using the library ‘vegan’ (https://vegandevs.github.io/vegan/). Alpha diversity for sample was assessed using Shannon index, and measure of biodiversity was evaluated using Richness Menhinick’s index. Shapiro–Wilk test was performed to evaluate the normality of distribution of data. Two-sided Student’s *t* test for independent samples and Mann–Whitney *U* test were performed on the calculated alpha diversity and biodiversity values on the basis of ‘P’ and ‘E’ categories. To identify possible sample stratification, beta diversity was assessed using Bray–Curtis index and Principal Coordinate Analysis (PCoA) was performed for each pair of categories. Analysis of variance test and Tukey’s honestly significant difference as post hoc test were carried out on the calculated beta diversity values. The statistical significance was set at 0.05.

## Results

All the crown samples tested negative in the sterility test. Bacterial DNA was identified in all the 12 samples analyzed from the study group and the control sample. Negative extraction controls (DMEM and reagents from the DNA extraction kit) did not result in library prep due to low DNA concentration and were not sequenced. Mock community control DNA included in the sequencing runs most closely approximated to the theoretical composition of the mock community.

**Figure 1.**
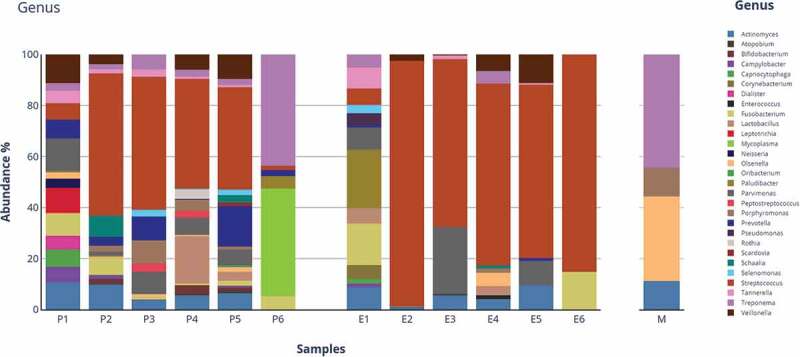
Sequence read distribution of bacterial genera detected in the periodontal (P), endodontic (E) and control (M) tissue samples of root canals from teeth of patients expressed as BarPlot charts.

**Figure 2.**
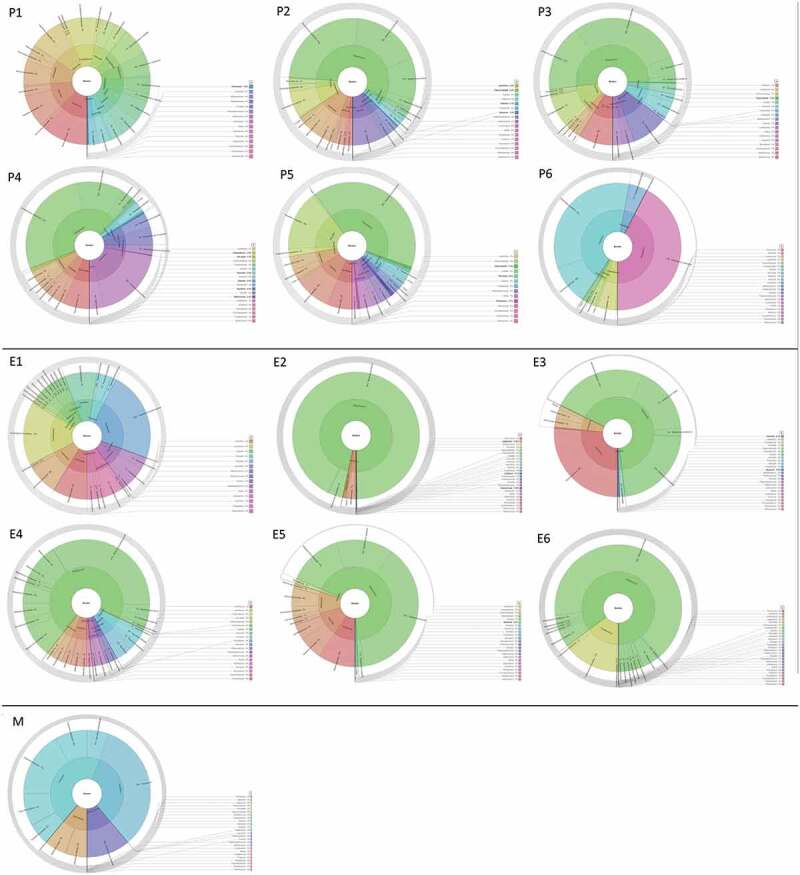
Sequence read distribution of bacterial species detected in the periodontal (P), endodontic (E) and control (M) tissue samples of root canals from teeth of patients expressed as multi-layered pie charts (Krona visualization tool).

After quality control of Nanopore sequence data, a total of 445,215 bacterial 16S rRNA gene sequence reads (mean, 37,101; median, 12,870; range, 3,255–23,0280) were obtained in the 12 analyzed samples but only 36 sequence reads in the control sample. A total of 123 Operational Taxonomic Units (OTUs) were identified in the P and E samples and assigned to 8 phyla, 29 genera and 86 species using Fastq 16S 2021.09.09 workflow. Overall, the most abundant phylum detected was *Bacillota* (80%) followed by *Actinomycetota* (6.1%) and *Bacteroidotes* (5.6%). The prominent genus was *Streptococcus* (72.9%) followed by *Veillonella* (3.6%), *Actinomyces* (3.6%), *Parvimonas* (3.5%), and *Prevotella* (3.1%) (Table 1, Figure 1) whilst *Streptococcus mutans* (50.7%) was the predominant species followed by *Streptococcus* a*nginosus* (6.3%) and *Parvimonas micra* (3.5%) (Figure 2).

**Table 1.** Distribution of sequence data, expressed as percentage, per bacterial genera in samples collected from periodontal (P) and endodontic (E) tissue samples from root canals of patients affected by severe periodontitis (Stage IV) according to the American Academy of Periodontology criteria.

Bacterial genera	Total reads (%)	P (%)	E (%)
*Actinomyces*	3.6	2.7	0.9
*Atopobium*	0.2	0.1	0.1
*Bifidobacterium*	0.7	0.7	0.0
*Campylobacter*	0.4	0.4	0.0
*Capnocytophaga*	0.1	0.1	0.0
*Corynebacterium*	0.1	0.1	0.0
*Dialister*	0.1	0.1	0.0
*Enterococcus*	0.1	0.1	0.0
*Fusobacterium*	1.6	1.4	0.2
*Lactobacillus*	1.8	1.7	0.1
*Leptotrichia*	0.2	0.2	0.0
*Mycoplasma*	0.6	0.6	0.0
*Neisseria*	0.1	0.1	0.0
*Olsenella*	0.4	0.4	0.0
*Oribacterium*	0.1	0.1	0.0
*Paludibacter*	0.2	0.1	0.1
*Parvimonas*	3.5	2.2	1.3
*Peptostreptococcus*	0.4	0.4	0.0
*Porphyromonas*	1.3	1.3	0.0
*Prevotella*	3.1	3.1	0.0
*Pseudomonas*	0.1	0.1	0.0
*Rothia*	0.3	0.3	0.0
*Scardovia*	0.2	0.2	0.0
*Schaalia*	1.3	1.3	0.0
*Selenomonas*	0.5	0.5	0.0
*Streptococcus*	72.9	18.2	54.7
*Tannerella*	0.7	0.6	0.1
*Treponema*	1.9	1.8	0.1
*Veillonella*	3.6	2.1	1.4
Total	100.0	41.0	59.0

Although the six collected teeth samples did not present neither clinically evident coronal leakages nor root damages, *Streptococcus* (54.7%) was predominant in E samples (Table 1), and it was also identified in P samples (18.2%) (Table 1, Figure 1).

The bacterial genera identified in the analyzed samples is reported in Table 2, whilst Table 3 shows the distribution of bacterial genera, expressed as percentage, in P and E tissue samples from the root canals. Overall, the most prevalent bacterial genera in the 12 collected samples were *Streptococcus* (100.0%, 12/12), *Actinomyces* (83.4%, 10/12), *Fusobacterium* (66.7%, 8/12), *Parvimonas* (66.7%, 8/12), *Prevotella* (66.7%, 8/12), *Tannerella* (66.7%, 8/12), *Treponema* (66.7%, 8/12), and *Veillonella* (66.7%, 8/12) (Tables 2 and 3).

**Table 2.** Presence of bacterial genera in periodontal (P) and endodontic (E) tissue samples of root canals from patients affected by severe periodontitis.

	Samples											
P/E	P1	P2	P3	P4	P5	P6	E1	E2	E3	E4	E5	E6
Patient	#1	#2	#2	#2	#2	#3	#1	#2	#2	#2	#2	#3
**Bacterial Genera**												
*Actinomyces*	+	+	+	+	+	-	+	+	+	+	+	-
*Atopobium*	-	+	-	+	+	-	-	+	+	-	-	-
*Bifidobacterium*	-	+	-	+	+	-	-	-	-	-	-	-
*Campylobacter*	+	+	+	-	+	-	+	-	-	-	-	-
*Capnocytophaga*	+	+	-	-	-	-	+	-	-	-	-	-
*Corynebacterium*	-	-	-	-	-	-	+	-	+	-	-	-
*Dialister*	+	-	-	-	-	-	-	-	-	-	-	-
*Enterococcus*	-	-	-	-	-	-	-	-	-	+	-	-
*Fusobacterium*	+	+	+	+	+	+	+	-	-	-	-	+
*Lactobacillus*	-	-	-	+	+	-	+	-	-	+	-	-
*Leptotrichia*	+	+	-	-	-	-	-	+	-	-	-	-
*Mycoplasma*	-	-	-	-	-	+	-	-	-	-	-	-
*Neisseria*	+	-	-	-	-	-	-	-	-	-	-	-
*Olsenella*	+	+	+	+	+	-	-	-	-	+	-	-
*Oribacterium*	-	-	-	-	+	-	-	-	-	-	-	-
*Paludibacter*	+	-	-	-	-	+	+	-	-	-	-	-
*Parvimonas*	+	+	+	+	+	-	+	-	+	-	+	-
*Peptostreptococcus*	-	-	+	+	-	-	-	-	-	-	-	-
*Porphyromonas*	-	+	+	+	+	-	-	-	-	+	-	-
*Prevotella*	+	+	+	+	+	+	+	-	-	-	+	-
*Pseudomonas*	-	-	-	-	-	-	+	-	-	-	-	-
*Rothia*	-	-	-	+	-	-	-	-	-	-	-	-
*Scardovia*	-	-	-	-	+	-	-	-	-	-	-	-
*Schaalia*	-	+	-	-	+	-	-	-	-	+	-	-
*Selenomonas*	-	-	+	+	+	-	+	+	-	-	-	-
*Streptococcus*	+	+	+	+	+	+	+	+	+	+	+	+
*Tannerella*	+	+	+	+	+	-	+	-	+	-	+	-
*Treponema*	+	+	+	+	+	+	+	-	-	+	-	-
*Veillonella*	+	+	-	+	+	-	-	+	+	+	+	-

**Note: +**: Presence; -: Absence; **•**: same patient.

**Table 3.** Distribution of bacterial genera, expressed as percentage, in periodontal (P) and endodontic (E) tissue samples of root canals from patients affected by severe periodontitis.

Bacterial genera	P (%)	E (%)
*Actinomyces*	41.7	41.7
*Atopobium*	25.0	16.7
*Bifidobacterium*	25.0	0.0
*Campylobacter*	33.4	8.3
*Capnocytophaga*	16.7	8.3
*Corynebacterium*	0.0	8.3
*Dialister*	8.3	0.0
*Enterococcus*	0.0	8.3
*Fusobacterium*	50.0	16.7
*Lactobacillus*	16.7	16.7
*Leptotrichia*	16.7	8.3
*Mycoplasma*	8.3	0.0
*Neisseria*	8.3	0.0
*Olsenella*	41.7	8.3
*Oribacterium*	8.3	0.0
*Paludibacter*	16.7	8.3
*Parvimonas*	41.7	25.0
*Peptostreptococcus*	16.7	0.0
*Porphyromonas*	33.4	8.3
*Prevotella*	50.0	16.7
*Pseudomonas*	0.0	8.3
*Rothia*	8.3	0.0
*Scardovia*	8.3	0.0
*Schaalia*	16.7	8.3
*Selenomonas*	25.0	16.7
*Streptococcus*	50.0	50.0
*Tannerella*	41.7	25.0
*Treponema*	50.0	16.7
*Veillonella*	33.4	33.3

Alpha diversity among the samples, calculated using Shannon index, ranged between 0.053 and 0.909 (mean, 0.564; median, 0.611), while the biodiversity value using Richness Menhinick’s index ranged between 0.010 and 0.239 (mean, 0.091; median, 0.076). Comparisons of alpha diversity and biodiversity values did not reach the thresholds of statistical significance (*p* > 0.05) for the considered categories.

**Figure 3.**
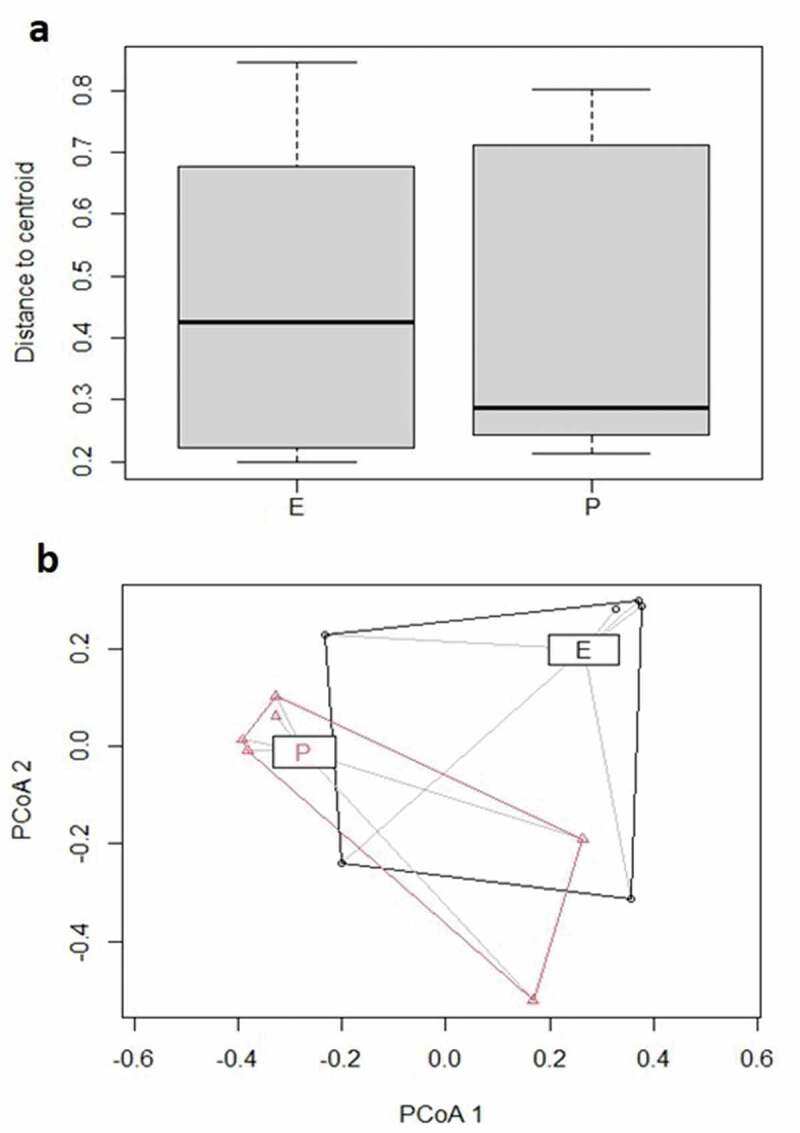
Bray– Curtis index (a) and Principal Coordinate Analysis (PCoA) (b) for periodontal (P) and endodontic (E) samples expressed as plots.

Beta diversity for P and E categories were assessed by using Bray–Curtis index and PCoA plot graphs were produced (Figure 3). Comparisons of beta diversity of samples did not reveal statistical significance (*p* > 0.05) for the categories.

The genera most frequently identified in P samples (*n* = 6) were *Fusobacterium* (50%), *Prevotella* (50.0%), *Streptococcus* (50.0%), *Treponema* (50.0%), *Actinomyces* (41.7%), *Olsenella* (41.7%), *Parvimonas* (41.7%), and *Tannerella* (41.7%). *Streptococcus* (50.0%) and *Actinomyces* (41.7%) were the most frequently detected genera in E samples (*n* = 6) (Tables 2 and 3). *Actinomyces* and *Streptococcus* were detected with equal prevalence (41.7–50.0%) in both P and E samples, whilst *Fusobacterium*, *Prevotella,* and *Treponema* were detected with higher prevalence in P (50.0%) than in E samples (16.7%) (Table 3).

*Atopobium* (41.7%, 5/12), *Selenomonas* (41.7%, 5/12), *Lactobacillus* (33.4%, 4/12), *Capnocytophaga* (25.0%, 3/12), *Leptotrichia* (25.0%, 3/12), *Paludibacter* (25.0%, 3/12), and *Schaalia* (25.0%, 3/12) were detected with equal or comparable prevalence in both P (16.7–25%) and E samples (8.3–16.7%). Conversely, *Campylobacter* (41.7%, 5/12), *Porphyromonas* (41.7%, 5/12), and *Bifidobacterium* (25.0%, 3/12) were identified more frequently in P samples (25.0–33.4%) than in E samples (0.0–8.3%) (Table 3). Multiple comparison between the obtained sequence reads in P and E samples revealed that the genera *Porphyromonas* (33.4% vs 8.3%, *p* = 0.047), *Tannerella* (41.7% vs 25%, *p* = 0.042), and *Treponema* (50.0%, *p* = 0.0064) were significantly more present in P than in E samples.

Samples P1 and P6 were collected from patient #1 and #3, respectively, and samples P2 to P5 were collected from patient #2. In the P samples collected from the three patients, yet in the context of an expected microbiome diversity, we observed the presence of the genera *Actinomyces, Fusobacterium, Olsenella, Parvimonas, Prevotella*, *Streptococcus*, *Tannerella,* and *Treponema*. Samples E6 and E1 exhibited a remarkable difference in terms of microbial composition, whilst the genus *Streptococcus* was a common signature in samples E2 to E5 obtained from the same patient (#2) (Table 2).

## Discussion

In this study, teeth affected by EPL Grade 3 with clinically intact crown’ surface and bone loss not reaching the apex were used. Therefore, a primary endodontic involvement was ruled out. The rationale for the study was based on the hypothesis that periodontal bacteria may reaches the root canal system even before the periodontal disease reaches the apical root segment.

In our study, all the teeth but the control case presented bacteria on the root surface and in the root canal system, demonstrating the possibility of bacteria to spread directly from the periodontal pocket to the root canal system, despite the apical foramen was not reached by periodontal lesion. It is well known that periodontal diseases may determine the exposure of other anatomical communications, over the apical segment, between periodontal tissues and the root canal system, with possible invasion of the root canal system from periodontal bacteria and/or their toxic metabolic products [18]. ‘Retrograde’ pulpitis is an inflammatory pulpal condition caused by response to bacterial invasion and toxic products entering through anatomical communications that became exposed to the oral fluids. Total pulp necrosis is determined when the blood supply to all root canals is interrupted by periodontal disease that involves apical root segment determining a vascular damage and subsequent hypoxia [1].

Alveolar bone resorption causes exposure of cementum that can be subsequently eroded mechanically, removed, and abraded with various processes. Loss of cementum exposes the dentinal tubules and allow bacterial entrance into the tooth. Moreover, the cementum–enamel junction (CEJ) presents a great morphological diversity, with frequent gaps between enamel and cementum with exposure of dentin, also among different surfaces of the same tooth [19]. Although in healthy teeth CEJ is normally protected and covered by gingival tissues (epithelial attachment and connective attachment) [20], it can be exposed for gingival recession and/or periodontal disease, with the possibility of an immediate exposure of dentinal tubules.

Lateral and accessory canals may distribute bacteria and toxins from the periodontal apparatus into the dental pulp [21,22].

Since they are located more at the coronal level than at the apical foramen, deep periodontal pockets can expose these communications without reaching the apex of the root. The control tooth in our study was also positive for bacterial DNA, revealing a low number of bacterial reads in the root canal system. This can suggest that pulpal tissue is not completely sterile also in the absence of deep periodontal pockets.

There are many controversial opinions regarding implications of severe periodontitis on inflammatory and degenerative alterations in the dental pulp. Some researchers suggest that periodontal disease can cause pulpal changes [23–26], whereas others do not [27,28].

Ricucci et al. evaluated teeth affected by periodontal disease with no clinically identified caries lesions, reporting histological and bacteriologic results consistent with bacterial colonization of the outer end of dentinal tubules when loss of integrity of the radicular cementum occurred. In some cases, the authors described histological aspects of pulpal degeneration due to bacterial colonization of the orifice of a lateral canal, with subsequent vascular damage and bacterial invasion of the pulpal bloodstream even before pulpal tissue necrosis [29]. These data support the results of this study, suggesting the possibility for periodontal bacteria to migrate in the root canal system also in absence of evident root damages, loss of crown integrity or massive periodontal disease reaching the apex.

Moreover, some bacteria seem to have a greater ability of migration between the two spaces. In fact, *Actinomyces*, *Streptococcus* [13], *Parvimonas,* and *Veillonella* were present with similar frequencies both in the periodontal pocket and in the root canal system.

Although their high frequency on root surfaces, some bacteria such as *Porphiromonas* and *Prevotella* were not identified in the root canal system. Several factors, including bacterial size, adhesive properties, motility, or micro-environmental selectivity may affect the degree of permeability to the dentinal tubules and virulence [19]

Overall, our study presents some limitations. The total sample size is relatively small and likely a larger sample size could be more useful to identify trends in the oral microbiome in these pathologies. Unfortunately, teeth affected by advanced periodontal disease without no clinically loss of external integrity and/or coronal leakages, which are ideal for similar studies, are not commonly observed in the clinical practice. In the presence of teeth affected by EPL with loss of crown’s integrity, there is an objective difficulty to establish if the primary bacterial involvement took place from the periodontal space or endodontic space.

Moreover, histologic investigations with bacterial staining were not carried out in our study to investigate the patterns of tissue invasion by bacteria in the endoperiodontal environments.

The study was based on teeth with poor prognosis, hindering any periodontal or endodontic treatment and with indication of extraction. It is unclear if endodontic contamination from periodontal space may occur also in teeth affected by less severe periodontitis.

The present study confirms the complexity of oral microbiome, organized in multispecies communities that may present important limits in microbiological evaluation using classic microbiological tests such as in vitro cultivation, typing with primers and probes and direct sequencing [30–32]. Massive sequencing techniques are a novel molecular method that may be applied to unveil the convoluted pictures of polymicrobial communities including low-abundance taxa or non-cultivatable species of oral microbiota [8,14,31,33–35]. In this study, we relied on a Nanopore 16s rDNA protocol to generate sequence data at a population level on the microbiological community. *16S rDNA* gene is a universal target for bacterial characterization with nine variable regions intermingled with conserved regions. Unlike other NGS techniques, Nanopore technology allows to generate sequence data on the full-length *16S rDNA* gene increasing the accuracy of characterization. Also, PCR-based enrichment with consensus primers allows to obtain data from biological matrices with low-density bacterial communities, for which otherwise, other sequencing approaches would not be feasible.

Considering the capacity of migration among periodontal and endodontic tissues, in case of surgical/non-surgical periodontal therapies and/or conservative rehabilitations of teeth affected by deep periodontal pockets, particular attention should be always paid to pulpal sensibility tests and pulpal symptoms to evaluate necrotic pulp or hyperresponsive vital pulp. In these cases, the root canal system should be considered as potentially contaminated by bacteria, and potentially acts as bacterial reservoir that may serve as recontamination source of residual pockets and/or periodontal tissues after surgical/non-surgical therapies [36], although not all bacterial species seem to possess the same capacity of migration. At the same time, an untreated deep periodontal pocket may serve as a source of periodontal bacteria to contaminate/re-contaminate the root canal system, determining pulpal/periapical pathology. Also, therapies that may remove cementum, such as root planning, should be carefully pondered for the potential exposure of dentinal tubules creating breaches for bacterial entry [28].

In conclusion, the results of the present study demonstrated the possibility of bacteria to spread directly from the periodontal pocket to the root canal system even in the absence of crown’s loss of integrity.

## Authors’ contributions

A.B. contributed to conception and design of the study, wrote the first draft of the manuscript; F.P. performed the experiments, organized the database, wrote the first draft of the manuscript; G.L. performed the statistical analysis and wrote the first draft of the manuscript; G.D. performed the experiments; M.S.L. performed the experiments; F.Z. wrote sections of the manuscript; M.C. reviewed and edited the manuscript; M.G.G. wrote sections of the manuscript; V.M. contributed to conception and design of the study, reviewed and edited the manuscript; C.P. contributed to conception and design of the study and reviewed and edited the manuscript. All authors contributed to manuscript revision and have read and approved the submitted version.

## Ethics approval and consent to participate

The study was performed in agreement with the ethical guidelines of the Declaration of Helsinki laid down in the 1964 and its later amendments or comparable ethical standards. The Ethics Committee of Azienda Unità Sanitaria Locale of Bologna approved this study with authorization no. 844-2021-OSS-AUSLBO-21160-ID 3118-Parere CE-AVEC-ENDO-MICROBIOTA 09/2021. Informed consent was obtained from all subjects and/or their legal guardian(s) for participation and publication.

## Availability of data and materials

All data generated or analyzed in this study are included in this published article.
